# Changes of Hematological and Hemorheological Parameters in Rabbits with Hypercholesterolemia

**DOI:** 10.3390/metabo11040249

**Published:** 2021-04-17

**Authors:** Bence Tanczos, Viktoria Somogyi, Mariann Bombicz, Bela Juhasz, Norbert Nemeth, Adam Deak

**Affiliations:** 1 Department of Operative Techniques and Surgical Research, Faculty of Medicine, University of Debrecen, Moricz Zsigmond u. 22, H-4032 Debrecen, Hungary; tanczos.bence@med.unideb.hu (B.T.); sogor.viktoria@med.unideb.hu (V.S.); nemeth@med.unideb.hu (N.N.); 2 Doctoral School of Clinical Medicine, University of Debrecen, Nagyerdei krt. 98, 4032 Debrecen, Hungary; 3 Department of Pharmacology and Pharmacotherapy, Faculty of Medicine, University of Debrecen, Nagyerdei krt. 98, 4032 Debrecen, Hungary; bombicz.mariann@pharm.unideb.hu (M.B.); juhasz.bela@med.unideb.hu (B.J.)

**Keywords:** hypercholesterolemia, rabbit model, hemorheology, atherosclerosis

## Abstract

Hypercholesterolemia plays an important role in the development of atherosclerosis, leading to endothelial dysfunction, ischemic events, and increased mortality. Numerous studies suggest the pivotal role of rheological factors in the pathology of atherosclerosis. To get a more detailed hematological and hemorheological profile in hypercholesterolemia, we carried out an experiment on rabbits. Animals were divided into two groups: the control group (Control) was kept on normal rabbit chow, the high-cholesterol diet group (HC) was fed with special increased cholesterol-containing food. Hematological parameters (Sysmex K-4500 automate), whole blood and plasma viscosity (Hevimet-40 capillary viscometer), red blood cell (RBC) aggregation (Myrenne MA-1 aggregometer), deformability and mechanical stability (LoRRca MaxSis Osmoscan ektacytometer) were tested. The white blood cell and platelet count, mean corpuscular volume, and mean corpuscular hemoglobin were significantly higher in the HC group, while the RBC count, hemoglobin, and hematocrit values were lower than the Control data. Viscosity values corrected to 40% hematocrit were higher in the HC group. The RBC aggregation significantly increased in the HC vs. the Control. The HC group showed significantly worse results both in RBCs’ deformability and membrane stability. In conclusion, the atherogenic diet worsens the hematological and macro- and micro-rheological parameters, affecting blood flow properties and microcirculation.

## 1. Introduction

Atherosclerosis is a generalized disease of the arterial wall, characterized by thickening of the intimal layer and accumulation of fat, partly caused by hyperlipidemia (high concentration of lipids and/or lipoproteins) and lipid oxidation (as low-density lipoprotein [LDL] oxidation) [1]. An increasing number of international multi-center studies (Edinburgh Artery Study, Puerto Rico Study, Caerphilly and Speedwell Study, Northwick Park Heart Study) have shown that the development of atherosclerosis and thrombotic predisposition is associated with changes in hemorheological factors [2,3,4,5]. There are several mechanisms by which hemorheological factors can promote atherogenesis. These include the hypercoagulability, which predisposes to thrombosis, the decreased blood flow due to rheological changes, and the increased concentration of fibrinogen and its metabolites [6,7].

The rabbit is a widely used animal model for the study of human metabolic diseases [8]. The lipid metabolism of rabbits makes these animals particularly suitable for the study of the pathophysiology of lipoprotein metabolism, atherosclerosis, and metabolic syndromes [9,10,11,12]. Furthermore, this species’ cardiac physiology (actin-myosin structure, ion channel characteristics) [13,14] is comparable to humans, defining the rabbit as an ideal model to study heart diseases as cardiac arrhythmia, myocardial infarction, heart failure, ischemic heart disease. A considerable number of articles have presented data about atherosclerotic rabbits [10,11], but the number of papers presenting detailed hematological and hemorheological data of healthy [15,16] and atherosclerotic [17] rabbits is relatively few.

The aim of our research was to evaluate how the atherogenic diet affects hematological, macro-rheological (whole blood cell and plasma viscosity) and micro-rheological (erythrocytes’ aggregation, deformability, and mechanical membrane stability) parameters in a rabbit model of cardiovascular disease.

## 2. Results

### 2.1. Bodyweight Changes

The bodyweight of the animals before starting the follow-up period with diet was 2898 ± 111 g in the Control and 2923 ± 133 g in the HC group. At the time of the blood sampling the weight of Control group animals was 3087 ± 56 g (*p* = 0.004 vs. base), and 4131 ± 61 g in the HC group (*p* = 0.002 vs. base, *p* < 0.001 vs. Control).

### 2.2. Hematological Parameters

Table 1 summarizes the hematological results. The white blood cell count, the mean corpuscular volume and platelet count were significantly higher in the HC group than in the Control group. Mean corpuscular hemoglobin did not differ significantly. The red blood cell count, the hemoglobin value and the mean corpuscular hemoglobin concentration significantly decreased in HC group versus to the Control group.

### 2.3. Hemorheological Parameters

#### 2.3.1. Whole Blood and Plasma Viscosity

**Figure 1:**
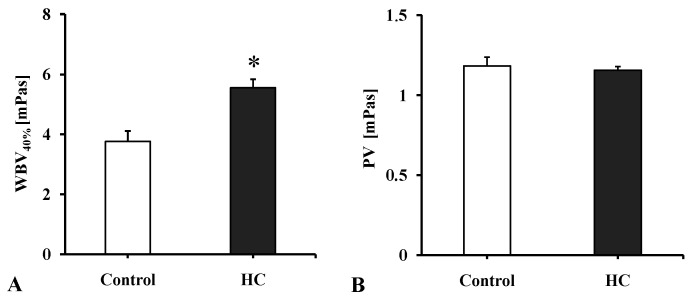
(**A**): whole blood viscosity corrected to 40% hematocrit (WBV [mPas]) and (**B**): plasma viscosity (PV [mPas]) values in the Control and the atherogenic groups (HC). Means ± SEM; * *p* < 0.05 vs. Control.

The whole blood viscosity values corrected to 40% hematocrit were significantly increased vs. the Control group’s data (*p* = 0.0051). In the plasma viscosity no considerable changes were detected (Figure 1).

#### 2.3.2. Red Blood Cell Aggregation

**Figure 2:**
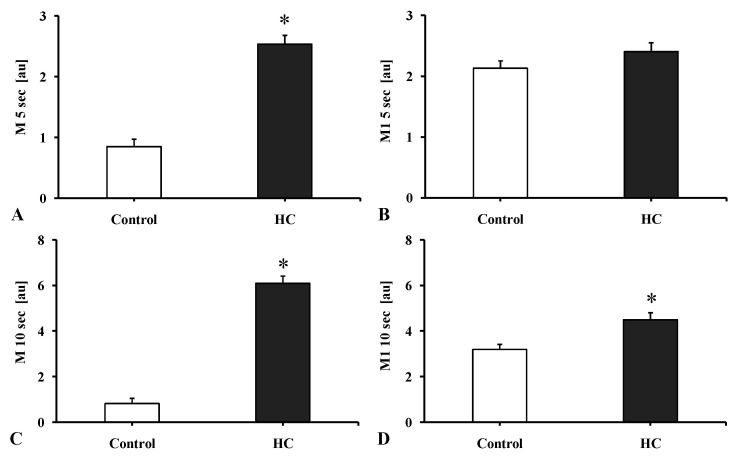
The red blood cell aggregation indices (**A**): M 5 s, (**B**): M1 5 s, (**C**): M 10 s, (**D**): M1 10 s [au]) measured by the Myrenne MA-1 aggregometer in the Control and the atherogenic group (HC). In M mode (shear rate = 0 s^−1^) and M1 mode (shear rate = 3 s^−1^) the index values are expressed at the 5th or at the 10th second of the aggregation. Means ± SEM; * *p* < 0.05 vs. Control.

In the HC group all the aggregation index values increased. The changes were significant at stasis (M 5 s: *p* < 0.001, M 10 s: *p* < 0.001) and at low shear rate (3 s^−1^, M1 10 s: *p* = 0.0251) (Figure 2).

**Figure 3:**
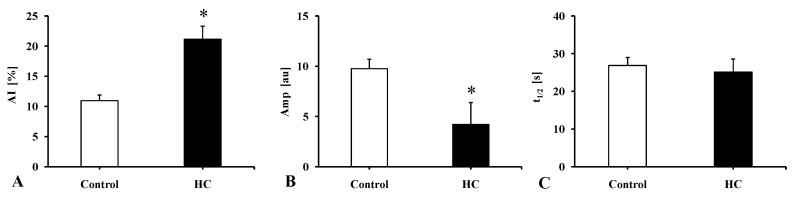
The red blood cell aggregation parameters measured by LoRRca rotational ectacytometer in the Control and the atherogenic group (HC). (**A**): aggregation index (AI [%]), (**B**): amplitude (Amp [au]) of the aggregation syllectogram (maximal-minimal intensity), (**C**): t_1/2_ [s] representing the aggregation time at half Amp. Means ± SEM; * *p* < 0.05 vs. Control.

Figure 3 shows the changes in erythrocyte aggregation values determined by the LoRRca device. The difference in aggregation index (AI [%]) values was highly significant (*p* = 0.0003) between Control vs. HC groups. The amplitude values (Amp [au]) were lower in the HC group than in the Control group (*p* < 0.0001). The t_1/2_ [s], which describes the kinetics of RBC aggregation, in the HC group values presented a non-significant decrease compared to the Control group.

#### 2.3.3. Red Blood Cell Deformability

**Figure 4:**
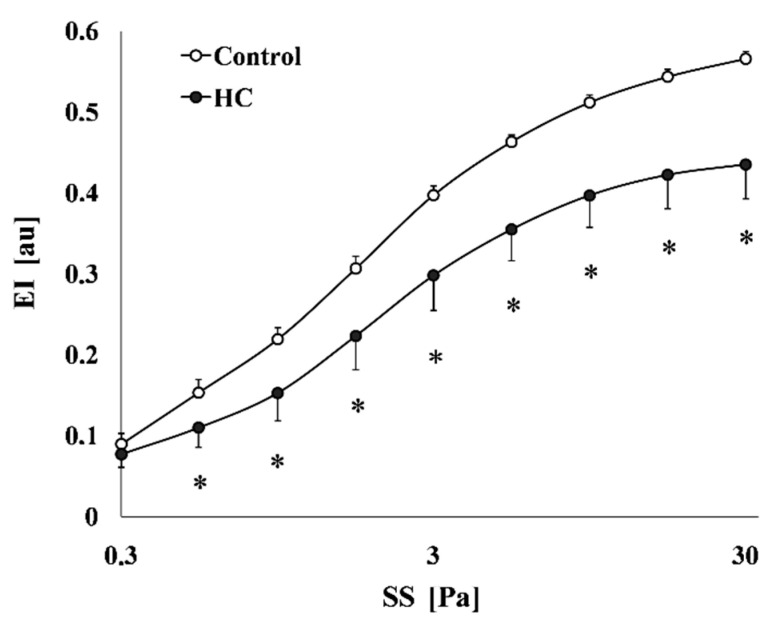
The red blood cell deformability describing elongation index (EI [au]) in the function of shear stress (SS [Pa]) of the Control and the atherogenic groups (HC); Means ± SEM; * *p* < 0.05 vs. Control.

The deformability of red blood cells was impaired in the HC group. The elongation index (EI)—shear stress (SS) curves showed remarkable differences, as the EI values of the HC groups were significantly lower compared to the Control group (Figure 4).

The calculated parameters from the individual EI-SS curves also expressed the differences. The EI_max_ data were higher in the Control group and the differences were significant versus the HC group. EI values at 3 Pa were significantly lower in the HC group. SS_1/2_ values were significantly increased in the HC group, while the EI_max_/SS_1/2_ ratio values of the HC group were lower than in the Control animals (Table 2).

#### 2.3.4. Red Blood Cell Membrane (Mechanical) Stability

**Figure 5:**
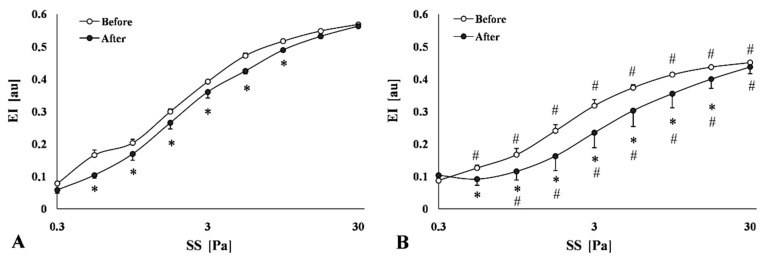
Elongation index (EI [au])—shear stress (SS [Pa]) curves in the mechanical stability test: before and after applying mechanical stress (100 Pa for 300 s) on the samples of the Control (**A**) and the atherogenic (HC) group (**B**). Means ± SEM; * *p* < 0.05 vs. before mechanical stress; # *p* < 0.05 vs. Control.

The EI-SS curves obtained before and after applied mechanical stress (100 Pa for 300 s) on the samples presented remarkable changes. Firstly, as the conventional deformability tests showed (see above), the HC group presented significantly lower EI values versus the Control with lower EI_max_ and higher SS_1/2_ values. Secondly, after the mechanical stress, the decrease in EI values was more expressed in the HC group. The decrease in EI and EI_max_ values, as well as the increase in SS_1/2_ values, were significant, compared to the Control group (Figure 5A,B, Table 3).

## 3. Discussion

The hypercholesterolemic rabbit model is a preferred model to study human atherosclerosis and lipoprotein metabolism [18,19]. It is well-known that rabbits are sensitive to dietary cholesterol and rapidly develop severe hypercholesterolemia which drives aortic atherosclerosis. The hepatic LDL receptors in both humans and rabbits are down-regulated according to the level of cholesterol uptake in the liver. Very-low-density lipoprotein (VLDL) receptors are highly expressed in macrophages, this is also a similarity between rabbits and humans [11]. The larger arteries compared to the small rodent models allow the clinical evaluation: using MRI and ultrasound (echocardiography), morphological (plaque composition and structure) and functional changes (systolic, diastolic dysfunction) can be detected [18,19]. The atherosclerotic rabbit model is considered suitable to investigate numerous human diseases, however, it presents several limitations: e.g., the laboratory rabbits do not develop spontaneous atherosclerosis on a standard diet, because they have low cholesterol levels. The severe pathological changes, such as worsened ejection fraction and general deterioration of cardiac functions, with increased atherosclerotic plaque formation, infarct size, and increased mortality, develop only in rabbits receiving high cholesterol diets for a long period of time [20]. When feeding the rabbits with high-cholesterol-containing food, aortic lesions can develop, first in the aortic arch and then in the thoracic aorta. The abdominal aortic lesions, characteristic for humans, appear only when the whole aortic lesions are severe. Coronary atherosclerosis is also observed in cholesterol-fed rabbits (with predilection in the left arterial trunks). Another disadvantage is that the advanced lesion as fibrosis, hemorrhage, ulceration, or aortic aneurysms are not seen; the rabbits’ plaque is characterized by foam cells with a fatty streak and they are rich in macrophages [11,18]. The so-called advanced lesions can develop following prolonged cholesterol feeding, but due to low hepatic lipase activity, this leads to increased hepatotoxicity [18].

In our study the used rabbit model was characterized by significant morphological, functional, and serological alterations. The area of the left atrium was enlarged; the weight of the left ventricle and relative wall thickness was increased. During the histological analysis, a foamy atherosclerotic plaque was observed on aortic sections, while in myocardial tissue interstitial fibrosis was determined. Symptoms of diastolic dysfunction were detected too. The serum lipid parameters, the atherogenic index, and ApoB/ApoA ratio were increased significantly in rabbits fed with additional 1% cholesterol and 1% saturated fat [19]. However, the limitations of the study include the low case number and the inter-species differences as mentioned above.

The atherogenic diet has affected numerous hematological parameters in our study. The red blood cell and the hemoglobin count were considerably decreased. This alteration was reported in experimental rabbits with high total cholesterol and increased LDL levels [21]. The low RBC and hemoglobin can appear even after 6 weeks of the experiment in rabbits, together with MCV, MCH, and MCHC changes [22,23]. In our investigation, in the HC group, the significantly increased MCV, unchanged MCH, and significantly decreased MCHC count show the signs of macrocytic hypochromic regenerative anemia. Anemia causes hypoxia due to decreased hemoglobin level, and there are several hemodynamic and non-hemodynamic compensatory mechanisms. The clinical and hemodynamic changes as a result of acute anemia are reversible, but chronic anemia drives progressive cardiac enlargement and left ventricular hypertrophy [24,25,26]. This cardiac alteration was detected in our rabbits, too [19]. The elevated MCV can be associated with the severity of atherosclerotic alterations and deficiency of vitamins related to atherosclerotic diseases as well [27].

Hypercholesterolemia stimulates platelet biogenesis through megakaryopoiesis, and leukocytosis by myelopoiesis, and increases platelet activation, by promoting platelet production and by direct impact on platelets [28,29,30,31]. The increased cholesterol level enhances the hyperaggregability of thrombocytes, too. Activated platelets can form aggregates with neutrophils and monocytes, and the subsequent crosstalk between platelets and leukocytes also plays an important role in the production of inflammatory cytokine, in the biosynthesis of leukotrienes and reactive oxygen species (ROS) [28]. The ROS can induce the production of inflammatory mediators such as C-reactive protein (CRP), which can activate the pro-thrombotic factors and platelets [32,33]. In our experimental animals, we detected significantly increased white blood cell and platelet count and CRP level [19]. These markers have shown the inflammatory character of atherosclerosis.

The high cholesterol level has direct effects on blood flow; this includes the growth of atherosclerotic plaques in the arterial system, reducing the lumen of coronary arteries, causing endothelial inflammation, and impaired endothelium-dependent vasorelaxation [34]. All together, these lead to an impairment of myocardial circulation and tissue perfusion [35]. Indirect effects of hypercholesterolemia involve blood rheology: a high level of cholesterol may increase whole blood viscosity by promoting the elevation of white blood cell and platelet count [29,36].

Remarkable changes in red blood cell aggregation using light-transmittance and syllectometry methods were observed. With light-transmittance, we detected that the HC group has significantly increased aggregation index values in each measuring mode (M and M1). Using the syllectometry method, in the HC group an increased aggregation index was accompanied by decreased aggregation amplitude, with unchanged time values. A similar tendency in aggregation index and syllectogram amplitude was reported in a clinical trial, performed with the same measuring method, on obese diabetic patients with hypercholesterolemia [37,38]. This study revealed that the total cholesterol level is correlated positively with the RBC aggregation index and negatively with aggregation half-time.

Red blood cell aggregation under low shear conditions is the main cause of increased blood viscosity [39,40,41,42]. The blood flow resistance during aggregation may decrease due to a reduced hematocrit but can increase at the same time by redistribution of red blood cells. Diminished blood flow can be caused by adhesion reactions between the blood cells and the endothelium of capillaries, such as the adhesion of white blood cells during inflammatory processes. Narrowing of the vessels due to atherosclerotic plaque can also adversely affect and increase cellular adhesion and flow resistance [43,44,45]. The increased aggregation can stimulate the axial migration of red blood cells which promotes plasma skimming. In this environment, a lower tissue hematocrit may cause decreased local viscosity at the marginal zone of blood vessels and could reduce the frictional resistance with the endothelium [46,47,48,49]. The axial migration promotes the phenomenon called margination described in white blood cells and platelets. The rigid RBC increases platelet marginalization which increases the tendency for thrombosis. Munn and Dupin [47] showed that the rouleaux formation of aggregating RBC is a more productive way to push the WBC to the vascular wall compared to a loosely associated group of cells. The WBC margination depends on the flow properties, axial migration of RBCs and RBC aggregates, local hematocrit as well as on blood cell deformability [48,49].

The mechanical stability of erythrocytes is essential to complete their function and to survive in the blood circulation. Several physiological and pathophysiological changes can affect the determining parameters of red blood cell deformability [45,50,51]. The stability of erythrocytes is directly related to LDL-cholesterol levels [52]. An excessive increase in the quantity of cholesterol in the erythrocyte membrane increases the rigidity and decreases the membrane deformability. This rigidity is correlated to the increase in relative cholesterol/phospholipid ratio [53]. The changes in cholesterol/phospholipid ratio can also affect RBCs’ phosphatidylserine by reducing the exposure on the external surface of the cell in patients with hypercholesterolemia and spur cell anemia (in vitro study [54]. In high-fat diet fed mice, the levels of membrane cholesterol and phosphatidylserine externalization were increased, promoting erythrocytes-macrophage inflammatory interactions, and promoting macrophage phagocytosis in vitro [55]. Impairment of red blood cell deformability in hypercholesterolemia has been shown in clinical cases as well [56]. The phosphatidylserine serves as a trigger for macrophage recognition for senescent cells and plays an important role in erythrocyte’s membrane stability [54,57].

Erythrocytes with excessively rigid membrane are less stable and more susceptible to lysis by mechanical forces especially when passing through narrow vessels (capillaries, spleen) [58,59]. In comparison to normal erythrocytes, the rigid ones are unable to deform well under shear stress, so the higher viscosity caused by increased shear force can also be attributed to impaired erythrocyte deformability [60]. Our result supports these findings: impaired deformability of red blood cells was accompanied by dramatically decreased membrane stability in the HC group. The examined sensitive parameters well expressed the differences in deformability (EI, EI_max_, SS_1/2_, and their ratio) and mechanical stability (before and after shear stress) deterioration of red blood cells [51]. The degradation of erythrocytes’ deformability in the HC group was confirmed by the parameterization of EI-SS curves and was manifested in a decrease of maximal RBC elongation index and higher SS_1/2_, and decreased SS_1/2_/EI_max_ values. The worsening of membrane stability was represented by EI-SS curves compared before and after mechanical stress application. We must remark that the HC group elongation index and the maximum of elongation have deteriorated significantly even before the application of the shear stress and this tendency exacerbates after the applied mechanical stress. This worsening was well presented by impaired SS_1/2_/EI_max_ ratio, too.

## 4. Materials and Methods

### 4.1. Experimental Animals

The animal experiments were approved by the University of Debrecen Committee of Animal Welfare and by the National Food Chain Safety Office (registration Nr.25/2013 UDCAW) in accordance with the national (Act XXVIII of 1998 on the protection and sparing of animals) and EU (Directive 2010/63/EU) regulations.

**Figure 6:**
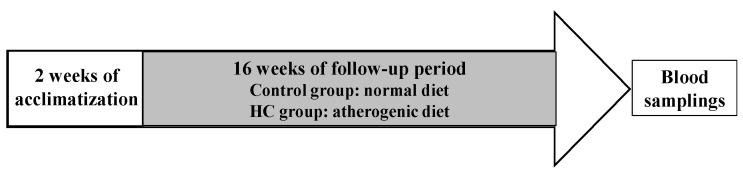
The timeline of the study.

Male Californian-New Zealand hybrid (CAL/NZW) rabbits (n = 12), age 20 weeks and 2700–3000 g bodyweight, were involved in this study. The animals were kept in a conventional experimental animal facility, under a 12 h-12 h light-dark cycle. The rabbits (Jurasko Ltd., Debrecen, Hungary) received during the first two weeks of the adaptation (acclimatization) period commercial laboratory rabbit chow. After the acclimatization period the animals were randomly divided into Control (n = 6) and high-cholesterol diet (HC), atherogenic group (n = 6). During the next 16 weeks, the animals of the Control group were fed with standard rabbit chow, while a special “atherogenic” chow (additional 1% cholesterol and 1% saturated fat, formulated in the Department of Pharmaceutical Technology, Faculty of Pharmacy, University of Debrecen) were given in the HC group [19,61]. At the end of the follow-up period, blood samples were taken (Figure 6).

### 4.2. Collection of Blood Samples

The blood samples were obtained from the marginal ear vein, with Vacutainer™-system, into a 3 mL BD Vacutainer^®^ tube containing 1.8 mg/mL K3-EDTA as anticoagulant (Becton, Dickinson and Company, Franklin Lake, NJ, USA) 2 mL blood samples per animal, and kept on 20 °C for further lab analysis. All laboratory measurements were completed within 2 h.

### 4.3. Laboratory Methods

#### 4.3.1. Hematological Parameters

A Sysmex K-4500 automate (TOA Medical Electronics Co., Ltd., Kobe, Japan) was used to determine the hematological parameters: red blood cell count (RBC [10^12^/µL]), white blood cell count (WBC [10^9^/µL]), hemoglobin concentration (Hgb [g/dL]), platelet count (Plt [10^9^/µL]). The hematocrit (Hct [%]), mean corpuscular volume (MCV [fL]), mean corpuscular hemoglobin (MCH [pg]), and mean corpuscular hemoglobin concentration (MCHC [g/L] were calculated from the measured data.

#### 4.3.2. Hemorheological Parameters

The changes in whole blood and plasma viscosity were measured by Hevimet-40 capillary viscometer (Hemorex Ltd., Budapest, Hungary) at 90 s^−1^ shear rates [16]. To calculate the whole blood viscosity the hematocrit count was normalized to 40% [62].

The erythrocytes’ aggregation was measured by light-transmittance method using Myrenne MA-1 erythrocyte aggregometer (Myrenne GmbH, Germany). After disaggregation (600 s^−1^) of 20 μL blood sample, at 5 or 10 s, the aggregation index values M mode (at a shear rate of 0 s^−1^) and M1 mode (at a shear rate of 3 s^−1^) were calculated [41,63]. The RBC aggregation was also tested with a LoRRca MaxSis Osmoscan ektacytometer (Mechatronics BV, The Netherlands). The device was operated with laser backscattering method. In the Couette-system, the blood sample is disaggregated by rotation, after this, the rotor stops promptly, and the changes in the intensity of the light reflected from the blood sample are measured [41,63]. The analyzed parameters were amplitude (Amp [au]), aggregation index (AI [%]) and half-amplitude time (t_1/2_ [s]). The test requires 1 mL of blood.

Using LoRRca MaxSis Osmoscan ektacytometer, for the deformability and membrane stability of erythrocytes, for each measuring, 10 μL of blood was diluted in 2 mL of polyvinyl-pyrrolidone (PVP)/phosphate-buffered saline (PBS) solution (viscosity: 36.1 mPas, osmolarity: 300, mOsm/kg, pH: 7.3). The elongation index (EI) values of red blood cells were tested in the function of shear stress (SS [Pa], range: 0.3–30 Pa) [41,63]. For the comparison of the EI-SS curves EI values at 3 Pa, maximal elongation index (EI_max_) and the shear stress belonging to the half of it (SS_1/2_, [Pa]) and their ratio (EI_max_/SS_1/2_) were used. These values were calculated using Lineweaver-Burk equation [64]. The cell membrane (mechanical) stability test was performed by comparing two deformability measurements before and after mechanical stress (100 Pa, for 300 s) [51,65].

### 4.4. Statistical Analysis

All data are presented as the average of the data on the group (mean) +/− standard error of the mean (SEM). The D’Agostino–Pearson normality test was used to determine Gaussian distribution, and statistical analysis was then performed using unpaired Student’s *t*-test or Mann–Whitney test (when normality test was not passed) between the groups, and two-way ANOVA was performed in case of the red blood cell deformability and membrane stability results. Analyses were carried out using GraphPad Prism software for Windows, version 8.0 (GraphPad Software Inc., La Jolla, CA, USA). Probability values (*p*) less than 0.05 were considered as statistically significantly different.

## 5. Conclusions

Macro- and micro-rheological parameters play an important role in determining tissue perfusion and shear stress-related endothelial functions and are influenced by numerous factors, involving metabolic changes too. Our study demonstrates that hypercholesterolemia can cause severe changes in hematological, macro-, and microrheological factors. The 16-week “atherogenic” diet altered not only the red blood cells’ number and hemoglobin content but also decreased the deformability and membrane stability of the erythrocytes. The aggregation indices of erythrocytes were characterized by a significant deterioration in the high cholesterol group, and this was proven by two different measuring techniques too. Our results may provide additional information to better understand the processes taking place in the vascular system during atherosclerosis and might contribute to optimizing the therapy.

## Figures and Tables

**Table 1:** Hematology parameters in the Control and the atherogenic groups (HC). Means ± SEM.

Hematological Parameter	Control (n = 6)	HC (n = 6)	*p* Value vs. Control
White blood cell count [×10^9^/L]	8.375 ± 0.270	23.59 ± 4.762	0.0042
Red blood cell count [×10^12^/L]	6.863 ± 0.125	3.758 ± 0.245	<0.0001
Hemoglobin [g/dL]	15.25 ± 0.272	8.392 ± 0.558	<0.0001
Hematocrit [%]	46.37 ± 0.929	29.38 ± 1.729	<0.0001
Mean corpuscular volume [fL]	67.58 ± 0.472	78.88 ± 2.426	0.0001
Mean corpuscular hemoglobin [pg]	22.22 ± 0.258	22.50 ± 0.757	n.s.
Mean corpuscular hemoglobin concentration [g/L]	32.90 ± 0.270	28.82 ± 1.270	0.0047
Platelet count [×10^9^/L]	254.8 ± 27.54	481.5 ± 38.73	<0.0001

**Table 2:** The red blood cell deformability measurements in the Control and the atherogenic groups (HC). EI at 3 Pa: elongation index at shear stress of 3 Pa, EI_max_: the maximal elongation index, SS_1/2_: shear stress belonging to the half of EI_max_. Mean ± SEM.

Parameter	Control	HC	*p* Value vs. Control
EI at 3Pa	0.591 ± 0.009	0.448 ± 0.051	<0.0001
EI_max_	0.397± 0.011	0.298 ± 0.043	<0.0001
SS_1/2_ [Pa]	1.544 ± 0.172	2.125 ± 0.633	0.0272
EI_max_/SS_1/2_ [Pa^−1^]	0.387 ± 0.044	0.228 ± 0.069	0.0007

**Table 3:** Comparative parameters of red blood cell membrane stability test before and after applying the mechanical stress (100 Pa for 300 s). EI at 3 Pa: elongation index at shear stress of 3 Pa, EI_max_: maximal elongation index, SS_1/2_: shear stress belonging to the half of EI_max_. Mean ± SEM.

Parameter	Test	Control	HC	*p* Value vs. Control, or vs. before (* Control, # HC)
EI at 3Pa	before (B)	0.392 ± 0.002	0.318 ± 0.015	0.002
	after (A)	0.360 ± 0.008 *	0.235 ± 0.019 #	0.0015; * 0.002; # 0.006
	ratio (A/B)	0.919 ± 0.019	0.734 ± 0.038	<0.0001
EI_max_	before (B)	0.592 ± 0.003	0.461 ± 0.017	<0.0001
	after (A)	0.595 ± 0.004	0.458 ± 0.011	<0.0001
	ratio (A/B)	1.005 ± 0.007	0.999 ± 0.033	ns
SS_½_ [Pa]	before (B)	1.690 ± 0.081	1.977 ± 0.227	ns
	after (A)	1.960 ± 0.149	4.540 ± 0.875 #	0.009; # 0.018
	ratio (A/B)	1.169 ± 0.089	2.228 ± 0.280	0.001
EI_max_/SS_1/2_ [Pa^−1^]	before (B)	0.355 ± 0.021	0.249 ± 0.028	0.012
	after (A)	0.311 ± 0.019	0.124 ± 0.025 #	0.0318; # 0.002
	ratio (A/B)	0.883 ± 0.062	0.484 ± 0.062	<0.0001

## Data Availability

The data presented in this study are available on request from the corresponding author.
